# A novel percutaneous transhepatic treatment of a benign bile duct stricture – a pilot study

**DOI:** 10.3325/cmj.2019.60.397

**Published:** 2019-10

**Authors:** Orsolya Huszár, Attila Szijarto, Tibor Tihanyi, László Harsányi, Ákos Szücs

**Affiliations:** 1st Department of Surgery, Semmelweis University, Budapest, Hungary

## Abstract

**Aim:**

To assess the effectiveness and outcome of repeated percutaneous transhepatic balloon dilatation combined with targeted intramucosal corticosteroid injection in patients with benign biliary stricture.

**Methods:**

This single-center pilot study, conducted between February 2014 and June 2016, involved five patients with benign biliary stricture (4 men and 1 woman, mean age 58.2 years). The study included only patients in whom previous surgical or/and non-surgical treatments failed or could not be performed due to patients’ medical history and local status.

**Results:**

We successfully developed an alternative treatment for patients with benign biliary stricture and performed it without side effects. There were no major complications, and the only one minor complication was cholangitis. In the median follow-up period of 30.24 months (range 14.5 to 44.6 months), no re-occlusion was detected. The disease-free survival, calculated after excluding the first patient (who died of heart attack), was 34.175 months.

**Conclusion:**

Percutaneous transhepatic corticosteroid injection combined with balloon dilatation could provide an alternative method for the treatment of benign biliary strictures that is effective in the long term and results in positive outcomes.

Benign biliary stricture is a rare condition mostly caused by operative trauma (1,2). The majority (80%) of surgical trauma is inflicted during cholecystectomy, with incidence rates of 0.2-0.3% after open cholecystectomy and 0.4%-0.6% after laparoscopic cholecystectomy (3). The rising number of laparoscopic cholecystectomies has increased the occurrence of benign biliary stricture. The second most common cause is fibrosis at the site of surgical anastomosis (1). Risk factors for stricture include anastomosis leakage, small stoma size, ischemia, and the stent placement at the stoma. Other causes are chronic pancreatitis, sclerosing cholangitis, cholelithiasis, impacted stones, sphincterotomy, and biliary tract infection (3,4).

The highest long-term stricture resolution rate was achieved by surgery (84%), followed by multiple plastic stent insertions (79%), and percutaneous transhepatic treatment with covered self-expandable metal stents (SEMS) (75%), without significant differences between the treatments (5). None of these therapies offer a definitive, reliable long-term solution for this disease, and therapy is still determined on a case-by-case basis. In addition, the long-term patency of these methods is unacceptable – particularly when considering the longer life expectancy of patients with this benign disease. All this indicates that benign biliary structures require new treatment strategies.

The pathophysiologic mechanisms involved in benign biliary stricture development are inflammatory responses with collagen deposition and fibrosis formation. Therefore, a viable treatment option could be pharmacological corticosteroid administration, especially as this method has been successfully used by plastic surgeons in scar therapy for almost 50 years (6). Since no data are available on percutaneous application at biliary strictures, we aimed to assess the effectiveness and outcome of repeated transhepatic balloon dilatation and corticosteroid injection in benign biliary strictures.

## Material and methods

This single-center pilot study, conducted between February 2014 and June 2016, involved five patients (4 men, 1 woman) with mean age of 58.2 years (range 32-74). The strict inclusion criteria: diagnosis of benign biliary stricture, contraindication or lack of feasibility of surgical and non-surgical (endoscopy) treatments due to general or local status, resulted in a super-selected patient group.

In three patients, benign biliary stricture occurred due to chronic pancreatitis and in two patients due to bile duct injury following laparoscopic cholecystectomy (Table 1). No surgical and non-surgical corrections of the anastomotic strictures had been permanently successful.

**Table 1:** Demographic and clinical data of patients with benign biliary stricture who underwent repeated percutaneous transhepatic balloon dilatation combined with targeted intramucosal corticosteroid injection

Patient No.	Age	Sex	Etiology	American Society of Anesthesiologists score	Previous surgery	Previous endoscopic retrograde cholangio-pancreatography	Bile duct stone
1	57	M	chronic pancreatitis	II	Frey’s procedure	yes*	yes
2	69	F	laparoscopic cholecystectomy	III	hepatico-jejunostomy	no^†^	yes
3	32	M	laparoscopic cholecystectomy	II	hepatico-jejunostomy	no^‡^	no
4	74	M	chronic pancreatitis	III	Frey’s procedure	yes^§^	yes
5	59	M	chronic pancreatitis	III	Frey’s procedure	no	yes

*Failed stent implantation.

†Failed balloon dilatations.

‡Insufficient and narrow anastomosis.

**§**Without long-term success.

All five patients underwent the same procedure complying with the ethical standards of Hungarian Medical Research Council (062350/2015/OTIG) and the Helsinki Declaration.

**Figure 1:**
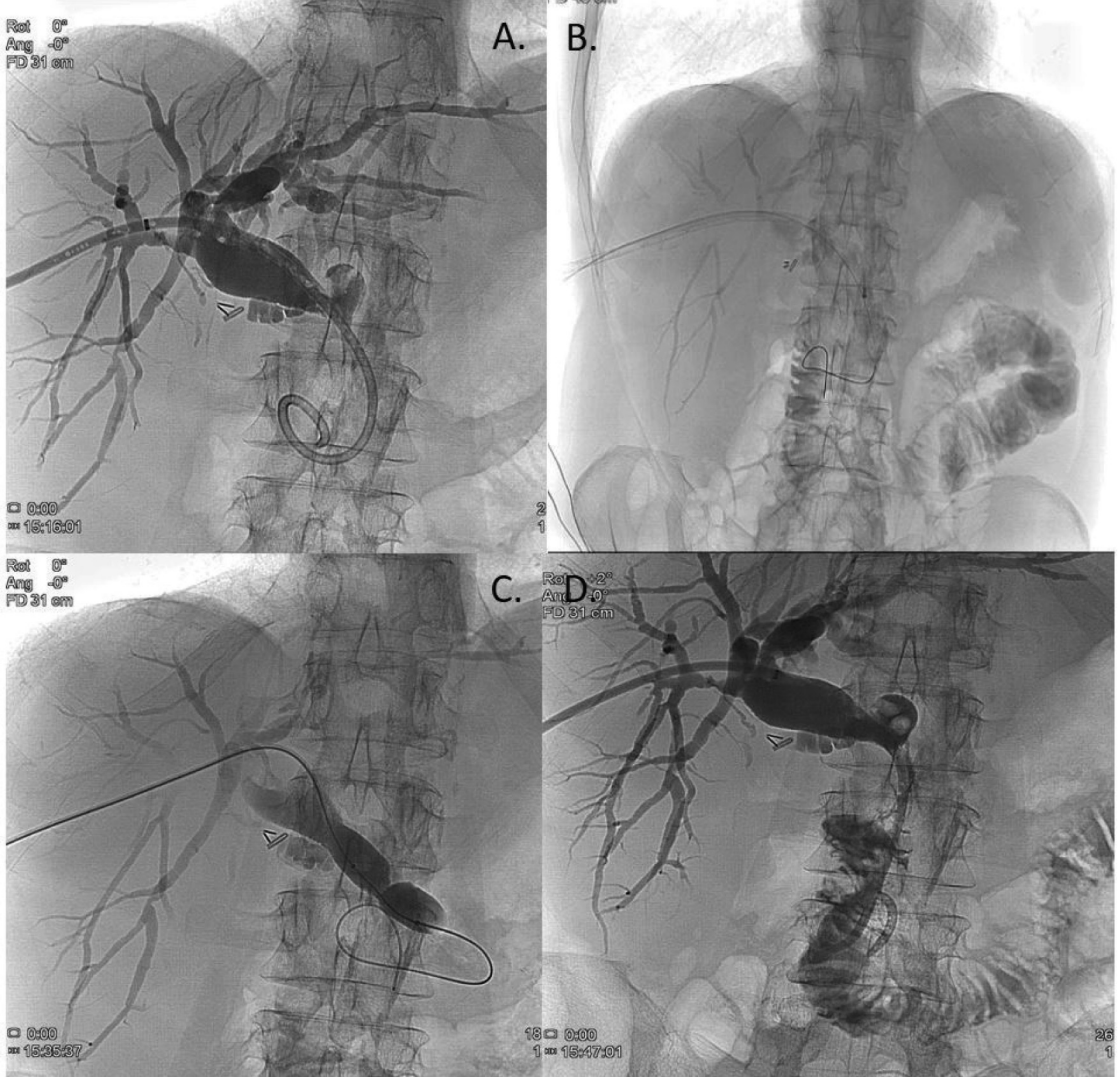
Technical steps of repeated percutaneous transhepatic balloon dilatation combined with targeted intramucosal corticosteroid injection. (**A**) Percutaneous transhepatic cholangiography. (**B**) Injection of 40 mg triamcinolone intramucosally into the stricture spatially with a flexible tip seeking catheter and a transluminal needle under fluoroscopic control. (**C**) Balloon dilatation (ø14 mm, 5 bar). (**D**) Drainage (ø10.2 F).

We began each treatment with the patient in the supine position. The primary puncture was performed in the 9th-10th intercostal space on the patient's right side. Once the Chiba needle (Cook Medical, Bloomington, IN, USA) was in the bile duct, as confirmed with cholangiography, a 0.018-inch wire (Cook Medical) was advanced and the needle was removed. A percutaneous access set (Cook Medical) with two sheaths and a metal cannula was used to introduce a cannula accepting a larger wire suitable for the planned intervention. After the coaxial tip was inserted into the bile duct using the 0.018-inch wire, the two inner components were removed, leaving the outer 4French (F) sheath behind. Cholangiography was performed to determine the obstruction level. A 4F biliary manipulation catheter (Cook Medical) was used to cross the obstructing lesion. The 0.018-inch wire was left in place to preserve the route for security reasons. By means of a 0.038-inch wire, a 7F Flexor access with curved tip configuration (Cook Medical) was inserted into the stricture. After removing the inner stiffener, a flexible 0.7 mm diameter needle (Endoflex Gmbh, Voerde, Germany) was introduced. Then 40 mg triamcinolone (Kenalog, KRKA d.d., Novo Mesto, Slovenia) was injected spatially into the stricture and equally distributed by manipulating the flexible tip seeking catheter and a transluminal needle under continuous fluoroscopic control. After the injection, balloon dilatation (14 mm in diameter) with 5-bar pressure was repeated three times. Finally, a 10.2F drain (Cook Medical) was left behind bridging the stricture (Figure 1, A-D).

This procedure was performed two more times with a one-month recovery period between treatments. The drains were removed two weeks after the third treatment. The follow up consisted of blood tests and ultrasound examinations every six months in the first year and blood tests and MR scans once each following year.

## Results

All patients tolerated their procedures well, and all 15 treatments were successful, with no technical failures or Kenalog-related side effects. Clinical success was achieved in all patients without major complications, such as perforation (bile leak), hemobilia, sepsis, pneumothorax, or death. Only one minor complication, cholangitis, occurred in the fourth patient due to drain occlusion, which was treated with simple drain lavage and conservative antibiotic therapy. Four patients had stone extraction (two percutaneously assisted during the last treatment, two after the treatments with rendezvous technique). One patient required an additional treatment one year after the procedure, when an elevated obstructive parameter (alkaline phosphatase: 468 U/L) indicated the presence of a non-symptomatic biliary stone. After this treatment, the patient developed cholangitis, but no new biliary stone presented during the follow up. Two patients complained of intermittent mild pain for one year after the procedure.

**Figure 2:**
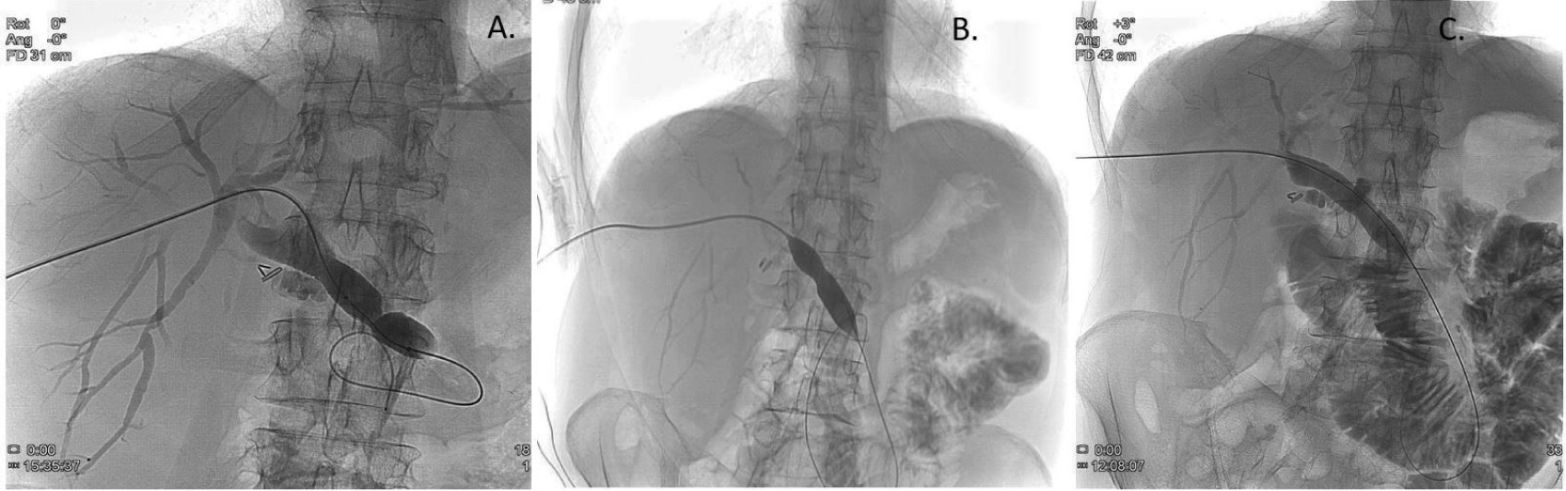
Results of the dilatations with intramucosal corticosteroid administration. Dilatation using ø14 mm balloon with 5 Bar as the first (**A**), second (**B**), and third (**C**) treatment. The increasing maximal extension of the balloon shows the radical resolution of the stricture.

Each intervention lasted on average 30-45 minutes. The inpatient length of stay averaged three days, and the patients were discharged home without any complaints. The control fluoroscopic images completed after the repeated procedures showed radical extension at each time point compared with the first dilatation (Figure 2, A-C).

The final follow-up visits took place in February 2018, when clinical examinations, blood tests, and ultrasound were performed. The median follow-up period was 30.24 months (range 14.5-44.6 months). The first patient died of a treatment-unrelated cause (heart attack) 14.5 months after the end of treatment and he was excluded from the disease-free calculation (Table 2). None of the patients had re-occlusion. After the end of the procedures, the patients gained from 3 to 25 kg (mean 7.6 kg). None of the patients reported pain or other biliary stricture related symptoms. The last ultrasound examinations showed no significant obstruction in any patient. The intrahepatic bile ducts and extrahepatic duct were in the reference range (Table 2). The disease-free survival was 34.175 months.

**Table 2:** Follow up results in patients with benign biliary stricture who underwent repeated percutaneous transhepatic balloon dilatation combined with targeted intramucosal corticosteroid injection

Patient No.	Period (month)	Conjugated bilirubin*	Alkaline phosphatase †	Imaging‡	Need for further intervention	Notes	Follow-up period (months)
1	6	within reference range	high	moderate intrahepatic bile duct dilatation, choledochus 7 mm, no stone, no bile duct obstruction	no	passed away from treatment non-related causes after 14.5 mo	14.5
	12	within reference range	within reference range	moderate intrahepatic biliary duct dilatation, no stone, no sign for bile duct obstruction			
2	6	within reference range	within reference range	no bile duct obstruction, no stone	no	completed the pilot study	44.6
	12	within reference range	high	no bile duct dilatation			
	24	within reference range	within reference range	no bile duct dilatation, no stone			
	36	within reference range	within reference range	moderate intrahepatic bile ducts, no stone			
	44.6	within reference range	within reference range	no bile duct dilatation, no stone			
3	6	within reference range	within reference range	no intrahepatic bile duct dilatation	no	completed the pilot study	33.6
	12	within reference range	within reference range	no bile duct dilatation, no stone			
	24	within reference range	within reference range	no bile duct dilatation, no stone			
	33.6	within reference range	within reference range	no bile duct dilatation	yes	non-symptomatic bile stone detected at 12 mo check-up without bile duct obstruction. We successfully performed the 4th treatment and stone extraction, but after this procedure a minor complication (cholangitis) occurred.	35.9
4	6	within reference range	within reference range	moderate bile duct dilation (better than previous), no stone			
	12	within reference range	high	choledocholithiasis, moderate (ductus choledochus 12 mm) bile duct dilatation			
	24	within reference range	within reference range	moderate dilated bile ducts, no stone, aerobilia			
	35.9	within reference range	within reference range	no intrahepatic bile duct dilatation, no stone			
5	6	within reference range	within reference range	no bile duct dilatation, no stone	no	completed the pilot study	22.6
	12	within reference range	within reference range	better dilatated bile duct dilatation, no stone			
	22.6	within reference range	within reference range	moderate bile duct dilatation, no stone			

*Reference range: 0-5.5 umol/L.

†Reference range: 80-300 U/L.

§Ultrasound, MRI, CT.

## Discussion

This study showed that our novel treatment could be a viable alternative solution for benign biliary stricture. Bile duct decompression can be achieved by endoscopy, percutaneous transhepatic intervention, or surgery. However, none of these approaches is generally accepted or yields a good long-term outcome, and the treatment is usually determined on a case-by-case basis (5).

Since non-surgical and surgical procedures have similar long-term patency rates, the selection of the treatment method varies based on several aspects, including the practitioner’s expertise. Furthermore, it is almost impossible to compare these methods due to unavailability of relevant long-term follow-up data (2-4). A multicenter, open-label, parallel, randomized clinical trial showed that covered SEMS had the highest resolution rate of 92.6% (7). Plastic stents had the highest resolution rate of 84%, which is unacceptable considering the benign nature of the disease with long life expectancy. Surgical approach aims to restore the bilio-digestive continuity with hepaticojejunostomy or choledochoduodenostomy. Hepaticojejunostomy is the preferable solution in patients with benign biliary strictures, however these patients are often poor candidates for surgery due to severe co-existing medical conditions such as malnutrition, cirrhosis, and portal hypertension (2,3,8).

Whereas biliary stents were formerly primarily used for the palliative treatment of malignant biliary obstructions, over the decades they have started to be used to treat benign biliary strictures. Single plastic stents – despite the easy applicability – have insufficiently small diameter and short patency. Therefore, there is a need for a more reliable multiple plastic stent, although even this type of stent would not be optimal because of short stent patency and the requisition of several endoscopic interventions. Other stents, such as cSEMS, have better stent-related conditions, but their reported long-term success rate is still 80%-90%. They also cause additional complications, like biliary infection, pancreatitis, bleeding, perforation, and early stent migration (4,7-10).

Another treatment, percutaneous biliary balloon dilatation, is safe, useful, and inexpensive for the treatment of the benign anastomotic stricture of Roux-en-Y hepaticojejunostomy. Furthermore, percutaneous biliary balloon dilatation repeated three times consecutively with fixed time interval yields the desired patency rate (11).

None of the five patients in this study were suitable for surgical treatment because of co-morbidities, intraoperative bleeding, or impracticability of the procedure. Due to their relatively young age (mean 58.2 years), they were also not suitable for biliary stent implantation since this procedure has unacceptable re-occlusion rate and inadequate long-term outcomes. This is why we sought for an alternative therapy that would provide good long-term outcome and would not lead to undesired effects of stenting or surgery.

The pathophysiologic mechanism involved in benign biliary stricture development is chronic inflammation accompanied with transmural fibrosis. The inflammatory process involving the massive aggregation of macrophages, synthesizing and secreting polypeptide growth factors, could induce a marked fibroblast proliferation and excessive synthesis and collagen deposition, resulting in a hypertrophic scar and consequential luminal obstruction (12). Meanwhile, plastic surgeons have used intralesional corticosteroid injection in scar therapy to decrease fibroblast proliferation, collagen synthesis, glycosaminoglycan synthesis, and suppress pro-inflammatory mediators (12). However, the procedure has widely variable response rates and recurrence rates (50%-100% and 9%-50%) (13), with a broad range of side effects, related only to prolonged usage due to the chemical nature of the drug, vehicle, and application site (14).

On the other hand, submucosal approach does not cause side effects (15-17), except with large doses of steroids and aggressive therapy, when systemic effects of triamcinolone acetonide could provoke iatrogenic Cushing syndrome due to adrenal insufficiency (18).

Several research groups have successfully applied corticosteroids to ameliorate different types of strictures. Ramage Jr et al (17) used them successfully in recalcitrant peptic esophageal stricture, which prolonged the need and the average time interval for repeat dilatations. Takahashi et al (19) showed profilactic endoscopic intramucosal steroid injection therapy to be safe and effective in the treatment of a stenosis caused by a mucosal defect involving the entire circumference of the esophagus after endoscopic submucosal dissection. Steroid injection could also be an effective and safe option in the treatment of therapy-resistant Lichen sclerosus (20) as well as of urethral strictures (12). However, in Crohn’s strictures intramural steroid injection could not be an option for adjuvant therapy (21). In 2010, an Italian randomized, double-blind, prospective pediatric study found that the endoscopic balloon dilatation with local corticosteroid injection successfully eliminated stricture and reduced the re-dilatation and surgery rate (16). The study finally concluded that for inflammatory bowel disease-related strictures, endoscopic balloon dilation, with or without topical corticosteroid injection, was a safe and effective intervention in patients with short, bland, symptomatic strictures, and may obviate surgery.

Newer antifibrotic agents, like mitomycin C, were introduced as a promising alternative to adjuvant treatment, but their efficacy has to be determined in further controlled trials. The intralesional injection of triamcinolone and topical mitomycin C showed better results in the treatment of benign esophageal strictures than balloon dilatation alone (22). Based on histopathologic evaluation, both mytomicin-C and triamcinolone decreased the recurrence rates of urethral stricture formation in New Zealand rabbits, with no significant differences between the agents (23).

There are no available data about percutaneous transhepatic intramucosal corticosteroid administration for the treatment of benign bile duct strictures. However, in 2015, Franzini et al (24) reported a successful balloon dilation with cholangioscopy-guided steroid injection for benign biliary stricture in a patient who developed an anastomosis stricture after orthotopic liver transplantation. Accordingly, after two sessions of biliary balloon dilatation and cholangioscopy-guided steroid injection, the patient recovered well without stent implantation and any adverse effects. Al Mahjoub et al (25) showed the clear superiority of the percutaneous approach compared with endoscopic interventions in malignant perihilar obstructions, with lower complication rate and less conversion. As our study showed, a definite advantage of the transhepatic approach is distinguished targeting and precise injection in the perihilar strictures. In special postsurgical cases, due to the modified anatomy, percutaneous transhepatic access may be the only possibility of stricture resolution.

Percutaneous transhepatic corticosteroid injection – as an alternative method – in combination with balloon dilatation could provide effective and long-lasting results in the treatment of benign pancreatogenic strictures. This novel method can be easily performed, is inexpensive and safe, and provides good quality of life for the patients. Although the present results are promising, due to the small number of patients involved, they need to be confirmed by further clinical and animal studies.
